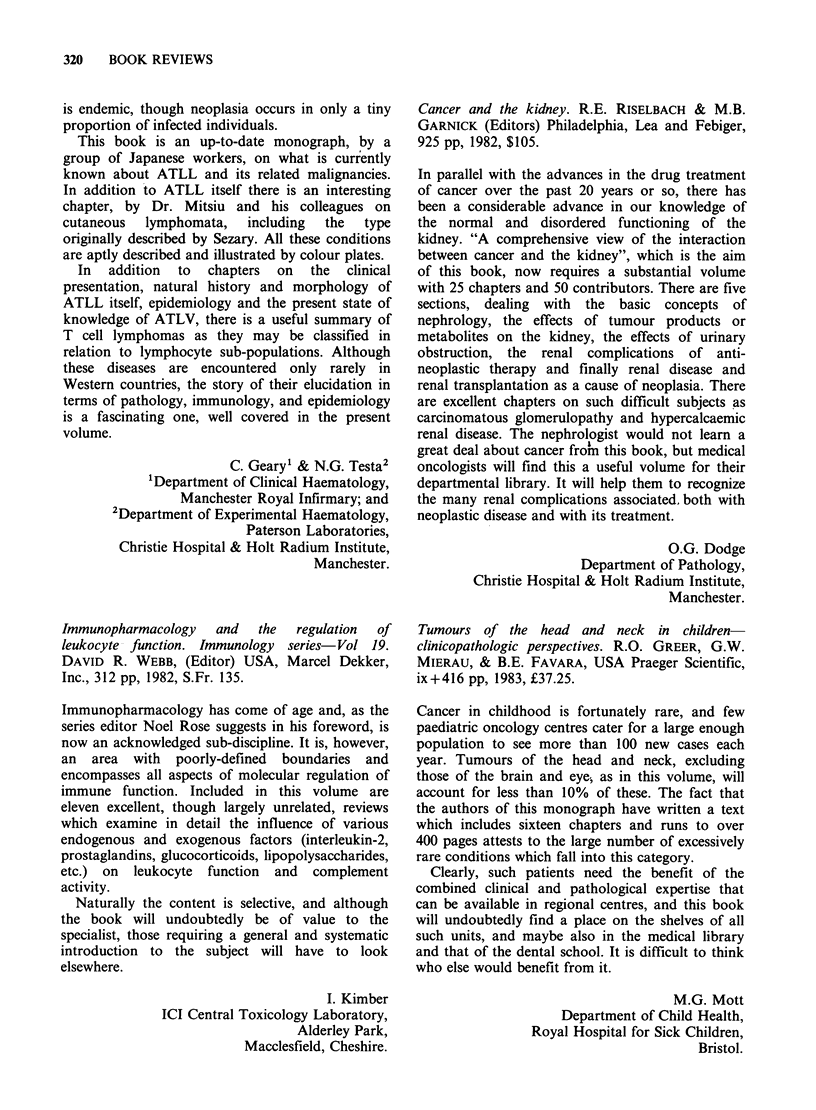# Tumours of the head and neck in children—clinicopathologic perspectives

**Published:** 1983-08

**Authors:** M.G. Mott


*Tumours of the head and neck in children—clinicopathologic perspectives.* R.O. Greer, G.W. Mierau, & B.E. Favara, USA Praeger Scientific, ix+416 pp, 1983, £37.25.

Cancer in childhood is fortunately rare, and few paediatric oncology centres cater for a large enough population to see more than 100 new cases each year. Tumours of the head and neck, excluding those of the brain and eye, as in this volume, will account for less than 10% of these. The fact that the authors of this monograph have written a text which includes sixteen chapters and runs to over 400 pages attests to the large number of excessively rare conditions which fall into this category.

Clearly, such patients need the benefit of the combined clinical and pathological expertise that can be available in regional centres, and this book will undoubtedly find a place on the shelves of all such units, and maybe also in the medical library and that of the dental school. It is difficult to think who else would benefit from it.

M.G. Mott
Department of Child Health,
Royal Hospital for Sick Children,
Bristol.